# Supplementary material for: Pharmacoinvasive strategy versus fibrinolytic therapy alone in adults with ST-elevation myocardial infarction: A systematic review and meta-analysis
Source: PLoS One. 2025 Oct 9;20(10):e0334309. doi: 10.1371/journal.pone.0334309 (PMC12510495; doi:10.1371/journal.pone.0334309)
Supplement: S2 Table — (DOCX) [file pone.0334309.s002.docx]

**Supplemental table 2. Search strategy**

Date of search: 17 of January 2025

| Database | Search strategy | Results |
| --- | --- | --- |
| Pubmed | #1 Myocardial infarction  "Myocardial Infarction"[Mesh] OR "Myocardial Infarct*"[tiab] OR “cardiac infarct*"[Tiab] OR “cardial infarct*"[Tiab] OR “heart attack*"[Tiab] OR “heart infarct*"[Tiab] OR “heart micro infarction*"[Tiab] OR “heart muscle infarction*"[Tiab] OR “myocardium infarct*"[Tiab] OR "ST Elevation Myocardial Infarction"[Mesh] OR STEMI[Tiab] OR “ST elevation MI”[TIAB] OR “ST elevated MI”[Tiab] OR “ST segment elevation MI” [Tiab]  #2 Pharmacoinvasive  “pharmaco-invasive”[Tiab] OR “pharmacoinvasive”[Tiab]  #3 Thrombolytic therapy  "Thrombolytic Therapy"[Mesh] OR Thromboly*[tiab] OR "Fibrinolytic Agents"[Mesh] OR Fibrinoly*[tiab]  #4 Percutaneous Coronary Intervention  "Percutaneous Coronary Intervention"[Mesh] OR "Percutaneous Coronary Intervention*"[tiab] OR "Percutaneous Coronary Revascularization*"[tiab] OR "Angioplasty, Balloon, Coronary"[Mesh] OR " Coronary Balloon Dilation"[tiab] OR “coronary artery dilatation"[tiab] OR "Angioplasty"[Mesh] OR "Angioplast*"[tiab] OR “Endoluminal Repair*”[tiab]    #5 Randomized clinical trials  "Randomized Controlled Trial"[PT] or "Randomized Controlled Trials as Topic"[Mesh] OR "Clinical Trial"[PT] OR "Clinical Trials as Topic"[Mesh] OR "Clinical Trial, Phase III"[PT] OR "Clinical Trial, Phase II"[PT] OR "Double-Blind Method"[Mesh] OR "Random Allocation"[Mesh] OR "Single-Blind Method"[Mesh] OR (random*[TIAB]) OR ((randomized[TIAB] OR randomised[TIAB] OR clinical[TIAB] OR control*[TIAB]) AND trial[TIAB]) OR ((singl*[TIAB] OR doubl*[TIAB] OR trebl*[TIAB] OR tripl*[TIAB]) AND (blind*[TIAB] OR mask*[TIAB]))  #1 AND (#2 OR (#3 AND #4)) AND #5 | 2990 |
| Web of Science | #1 Myocardial infarction  TI=("Myocardial Infarct*" OR “cardiac infarct*" OR “cardial infarct*" OR “heart attack*" OR “heart infarct*" OR “heart micro infarction*" OR “heart muscle infarction*" OR “myocardium infarct*" OR "ST Elevation Myocardial Infarction" OR STEMI OR “ST elevation MI” OR “ST elevated MI” OR “ST segment elevation MI”) OR AB=("Myocardial Infarct*" OR “cardiac infarct*" OR “cardial infarct*" OR “heart attack*" OR “heart infarct*" OR “heart micro infarction*" OR “heart muscle infarction*" OR “myocardium infarct*" OR "ST Elevation Myocardial Infarction" OR STEMI OR “ST elevation MI” OR “ST elevated MI” OR “ST segment elevation MI”)  #2 Pharmacoinvasive  TI=(“pharmaco-invasive” OR “pharmacoinvasive”) OR AB=(“pharmaco-invasive” OR “pharmacoinvasive”)  #3 Thrombolytic therapy  TI=("Thrombolytic Therapy" OR Thromboly* OR "Fibrinolytic Agents" OR Fibrinoly*) OR AB=("Thrombolytic Therapy" OR Thromboly* OR "Fibrinolytic Agents" OR Fibrinoly*)  #4 Percutaneous Coronary Intervention  TI=("Percutaneous Coronary Intervention" OR "Percutaneous Coronary Intervention*" OR "Percutaneous Coronary Revascularization*" OR "Angioplasty, Balloon, Coronary" OR " Coronary Balloon Dilation" OR “coronary artery dilatation" OR "Angioplasty" OR "Angioplast*" OR “Endoluminal Repair*”) OR AB=("Percutaneous Coronary Intervention" OR "Percutaneous Coronary Intervention*" OR "Percutaneous Coronary Revascularization*" OR "Angioplasty, Balloon, Coronary" OR " Coronary Balloon Dilation" OR “coronary artery dilatation" OR "Angioplasty" OR "Angioplast*" OR “Endoluminal Repair*”)  #5 Randomized clinical trials  TI=("Randomized Controlled Trial" or "Randomized Controlled Trials as Topic" OR "Clinical Trial" OR "Clinical Trials as Topic" OR "Clinical Trial, Phase III" OR "Clinical Trial, Phase II" OR "Double-Blind Method" OR "Random Allocation" OR "Single-Blind Method" OR (random*) OR ((randomized OR randomised OR clinical OR control*) AND trial) OR ((singl* OR doubl* OR trebl* OR tripl*) AND (blind* OR mask*))) OR AB=("Randomized Controlled Trial" or "Randomized Controlled Trials as Topic" OR "Clinical Trial" OR "Clinical Trials as Topic" OR "Clinical Trial, Phase III" OR "Clinical Trial, Phase II" OR "Double-Blind Method" OR "Random Allocation" OR "Single-Blind Method" OR (random*) OR ((randomized OR randomised OR clinical OR control*) AND trial) OR ((singl* OR doubl* OR trebl* OR tripl*) AND (blind* OR mask*)))  #1 AND (#2 OR (#3 AND #4)) AND #5 | 1999 |
| Embase | #1 Myocardial infarction    (‘heart infarction’ OR ‘ST segment elevation myocardial infarction’)/exp OR (STEMI OR ‘ST elevation MI’ OR ‘ST elevated MI’ OR ‘ST segment elevation MI’):ti,ab,kw OR ((myocard* OR heart OR cardia*) NEAR/2 (infarct* OR attack*)):ti,ab,kw  #2 Pharmacoinvasive  (‘pharmaco-invasive’ OR ‘pharmacoinvasive’):ti,ab,kw  #3 Thrombolytic therapy  (‘fibrinolytic therapy’ OR ‘fibrinolytic agent’)/exp OR (Thromboly* OR Fibrinoly*):ti,ab,kw  #4 Percutaneous Coronary Intervention  (‘percutaneous coronary intervention’ OR angioplasty)/exp OR (‘Percutaneous Coronary’ NEAR/2 (Intervention* OR Revascularization*)):ti,ab,kw OR (‘Coronary Balloon Dilation’ OR ‘coronary artery dilatation’ OR ‘Angioplast*’ OR ‘Endoluminal Repair*’):ti,ab,kw  #5 Randomized clinical trials  (‘randomized controlled trial’ OR ‘clinical trial’)/exp OR ((randomized OR randomised OR clinical OR control*) NEAR/2 trial):ti,ab,kw OR ((singl* OR doubl* OR trebl* OR tripl*) NEAR/2 (blind* OR mask*)):ti,ab,kw  #1 AND (#2 OR (#3 AND #4)) AND #5 | 3807 |
| CENTRAL | 1 Myocardial Infarction.sh.  2 ST Elevation Myocardial Infarction.sh.  3 (STEMI or ST elevation MI or ST elevated MI or ST segment elevation MI or ((myocard* or heart or cardia*) and (infarct* or attack*))).ab,ti.  4 (pharmaco-invasive or pharmacoinvasive).ab,ti.  5 Thrombolytic Therapy.sh.  6 Fibrinolytic Agents.sh.  7 (Thromboly* or Fibrinoly*).ab,ti.  8 Percutaneous Coronary Intervention.sh.  9 Angioplasty, Balloon, Coronary.sh.  10 ((Percutaneous Coronary and (Intervention* or Revascularization*)) or Coronary Balloon Dilation or coronary artery dilatation or Angioplast* or Endoluminal Repair*).ab,ti.  #11 (#1 OR #2 OR #3) AND (#4 OR ((#5 OR #6 OR #7) AND (#8 OR #9 OR #10) | 1796 |
| Clinicaltrial.gov | #1 Myocardial infarction  Myocardial Infarct* OR cardiac infarct* OR cardial infarct* OR heart attack* OR heart infarct* OR heart micro infarction* OR heart muscle infarction* OR myocardium infarct* OR STEMI OR ST elevation MI OR ST elevated MI OR ST segment elevation MI  #2 Intervention  (pharmaco-invasive OR pharmacoinvasive) OR ((Thromboly* OR Fibrinoly*) AND (Percutaneous Coronary Intervention* OR Percutaneous Coronary Revascularization* OR Coronary Balloon Dilation OR coronary artery dilatation OR Angioplast*))  #1 AND #2 | 10 |
