# Supplementary material for: Pharmacoinvasive strategy versus fibrinolytic therapy alone in adults with ST-elevation myocardial infarction: A systematic review and meta-analysis
Source: PLoS One. 2025 Oct 9;20(10):e0334309. doi: 10.1371/journal.pone.0334309 (PMC12510495; doi:10.1371/journal.pone.0334309)
Supplement: S3 Table — (DOCX) [file pone.0334309.s003.docx]

**Supplemental table 3. Search strategy for minimal important differences (MID).**

Date of search: 28 of august 2025

| Database | Search strategy | Results |
| --- | --- | --- |
| PubMed | #1 Myocardial infarction  "Myocardial Infarction"[Mesh] OR "Myocardial Infarct*"[tiab] OR “cardiac infarct*"[Tiab] OR “cardial infarct*"[Tiab] OR “heart attack*"[Tiab] OR “heart infarct*"[Tiab] OR “heart micro infarction*"[Tiab] OR “heart muscle infarction*"[Tiab] OR “myocardium infarct*"[Tiab] OR "ST Elevation Myocardial Infarction"[Mesh] OR STEMI[Tiab] OR “ST elevation MI”[TIAB] OR “ST elevated MI”[Tiab] OR “ST segment elevation MI” [Tiab]  #2 Minimal important difference  "Minimal Clinically Important Difference"[Mesh] OR (minim*[tiab] AND important[tiab] AND difference*[tiab]) | 130 |
