# Supplementary material for: Pharmacoinvasive strategy versus fibrinolytic therapy alone in adults with ST-elevation myocardial infarction: A systematic review and meta-analysis
Source: PLoS One. 2025 Oct 9;20(10):e0334309. doi: 10.1371/journal.pone.0334309 (PMC12510495; doi:10.1371/journal.pone.0334309)
Supplement: S4 Table — (DOCX) [file pone.0334309.s004.docx]

**Supplemental Table S4. Excluded studies reviewed at full text stage**

| Author (year) | Title | Exclusion reasons |
| --- | --- | --- |
| TIMI study group (1989) | Comparison of invasive and conservative strategies after treatment with intravenous tissue plasminogen activator in acute myocardial infarction. Results of the thrombolysis in myocardial infarction (TIMI) phase II trial | No stent |
| SWIFT trial study group (1991) | SWIFT trial of delayed elective intervention v conservative treatment after thrombolysis with anistreplase in acute myocardial infarction. SWIFT (Should We Intervene Following Thrombolysis?) Trial Study Group | No stent |
| De Luca (2005) | Tenecteplase followed by immediate angioplasty is more effective than tenecteplase alone for people with STEMI | Different publication type |
| ASSENT-4 PCI investigators (2006) | Primary versus tenecteplase-facilitated percutaneous coronary intervention in patients with ST-segment elevation acute myocardial infarction (ASSENT-4 PCI): randomised trial | Different comparator |
| Cantor (2008) | Trial of Routine Angioplasty and Stenting After Fibrinolysis to Enhance Reperfusion in Acute Myocardial Infarction (TRANSFER-AMI) | Different publication type |
| Agati (2007) | Does coronary angioplasty after timely thrombolysis improve microvascular perfusion and left ventricular function after acute myocardial infarction? | Different outcome |
| Armstrong (2020) | The Second Strategic Reperfusion Early After Myocardial Infarction (STREAM-2) study optimizing pharmacoinvasive reperfusion strategy in older ST-elevation myocardial infarction patients | Different comparator |
| Arnold (1992) | Recombinant tissue-type plasminogen activator and immediate angioplasty in acute myocardial infarction: One-year follow-up | No stent |
| Bagai (2014) | Routine invasive management early after fibrinolysis: relationship between baseline risk and treatment effects in a pooled patient-level analysis of 7 randomized controlled trials | Different study design |
| Bakal (2013) | Evaluation of early percutaneous coronary intervention vs. Standard therapy after fibrinolysis for ST-segment elevation myocardial infarction: contribution of weighting the composite endpoint | Different publication type |
| Barbash (1990) | Randomized controlled trial of late in-hospital angiography and angioplasty versus conservative management after treatment with recombinant tissue-type plasminogen activator in acute myocardial infarction | No stent |
| Belenkie (1992) | Rescue angioplasty during myocardial infarction has a beneficial effect on mortality: A tenable hypothesis | No full-text |
| Berry (2018) | A randomized, double blind, placebo-controlled, parallel group, multicenter clinical trial of low-dose adjunctive alteplase during primary PCI (T-TIME) | Different publication type |
| BÃ¸hmer (2007) | The norwegian study on district treatment of ST-elevation myocardial infarction (NORDISTEMI) | Different outcome |
| Bohmer (2009) | Immediate angioplasty compared to standard therapy after thrombolysis for ST-elevation myocardial infarction in areas with very long transfers. Results of the NORDISTEMI study | Different publication type |
| Buckley (2010) | Percutaneous Coronary Intervention After Successful Fibrinolytic Therapy for ST-Segment Elevation Myocardial Infarction. Better Late Than Never | Different publication type |
| Cantor (2006) | Transfer for urgent percutaneous coronary intervention early after thrombolysis for ST-elevation myocardial infarction: The TRANSFER-AMI pilot feasibility study | Different study design |
| Cantor (2008) | Rationale and design of the Trial of Routine angioplasty and Stenting After Fibrinolysis to Enhance Reperfusion in Acute Myocardial Infarction (TRANSFER-AMI) | Different study design |
| Cantor (2008) | ACCEL: Trial of Routine angioplasty and Stenting after Fibrinoiysis to Enhance Reperfusion in Acute Myocardial Infarction (TRANSFER-AMI) | No full-text |
| Chen (2021) | Rationale and design of the OPTIMAL-REPERFUSION trial: A prospective randomized multi-center clinical trial comparing different fibrinolysis-transfer percutaneous coronary intervention strategies in acute ST-segment elevation myocardial infarction | Different comparator |
| Czarnecki (2012) | Reperfusion strategies and outcomes of ST-segment elevation myocardial infarction patients in Canada: observations from the Global Registry of Acute Coronary Events (GRACE) and the Canadian Registry of Acute Coronary Events (CANRACE) | Different study design |
| Vyshlov (2018) | In-hospital and long-term results of delayed percutaneous coronary intervention after successful thrombolytic therapy in elderly patients with acute myocardial infarction | Different study design |
| Zavolozhin (2006) | [Effect of delayed coronary angioplasty after thrombolytic therapy on outcome of acute myocardial infarction] | No full-text |
| Itoh (2010) | Comparison of long-term prognostic evaluation between pre-intervention thrombolysis and primary coronary intervention: a prospective randomized trial: five-year results of the IMPORTANT study | Different intervention |
| Mehta (1994) | Sion thrombolysis trial--randomised trial of intravenous thrombolysis & primary percutaneous transluminal coronary angioplasty for acute myocardial infarction [AMI]--feasibility phase data of angioplasty limb | Different intervention |
| Meyerovitz (1995) | Thrombolytic therapy compared with mechanical recanalization in non-acute peripheral arterial occlusions: a randomized trial | Different population |
| Mihatov (2004) | Thrombolytic therapy or percutaneous coronary intervention in acute myocardial infarction | Different intervention |
| Mistry (2010) | Left ventricular function in acute myocardial infarction treated with thrombolysis followed by early versus late invasive strategy | Different intervention |
| Nagao (2002) | An early and complete reperfusion strategy for acute myocardial infarction using fibrinolysis and subsequent transluminal therapy--The FAST trial | No stent |
| Nct (2005) | Thrombolysis Versus Primary Angioplasty for AMI in Elderly Patients | Different intervention |
| Wexler (2001) | Non-Q-wave myocardial infarction following thrombolytic therapy: a comparison of outcomes in patients randomized to invasive or conservative post-infarct assessment strategies in the Veterans Affairs non-Q-wave Infarction Strategies In-Hospital (VANQWISH) Trial | Different population |
| Williams (1992) | One-year results of the Thrombolysis in Myocardial Infarction investigation (TIMI) Phase II Trial | Different intervention |
| Zhang (2009) | Comparative efficacy of primary percutaneous coronary intervention, facilitated percutaneous coronary intervention and fibrinolysis in Chinese patients with ST-elevation myocardial infarction: A multicenter randomized clinical trial | Different publication type |
| De Luca (2008) | The CARESS-in-AMI trial | No full text |
| Dieker (2006) | Transport for abciximab facilitated primary angioplasty versus on-site thrombolysis with a liberal rescue policy: the randomised Holland Infarction Study (HIS) | Different intervention |
| Dimopoulos (2012) | Timing of events in STEMI patients treated with immediate PCI or standard medical therapy: implications on optimisation of timing of treatment from the CARESS-in-AMI trial | Different population |
| Ellis (1994) | Randomized comparison of rescue angioplasty with conservative management of patients with early failure of thrombolysis for acute anterior myocardial infarction | Different population |
| Ellis (1992) | Randomized trial of late elective angioplasty versus conservative management for patients with residual stenoses after thrombolytic treatment of myocardial-infarction | Dftraifferent population |
| Groch (2003) | The 4-year clinical and angiographic follow-up in patients with acute myocardial infarction treated with immediate thrombolysis versus primary angioplasty versus combined strategy: Single-center randomized trial, subgroup of PRAGUE study | Different publication type |
