# Supplementary material for: Pharmacoinvasive strategy versus fibrinolytic therapy alone in adults with ST-elevation myocardial infarction: A systematic review and meta-analysis
Source: PLoS One. 2025 Oct 9;20(10):e0334309. doi: 10.1371/journal.pone.0334309 (PMC12510495; doi:10.1371/journal.pone.0334309)

**Supplemental Figure 2. Subgroup analyses**

1. Cardiogenic shock by risk of bias.


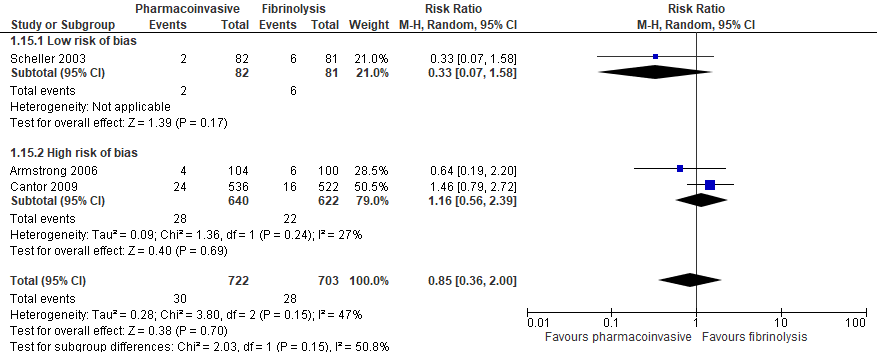


1. Recurrent ischemia (follow-up: 30 days) by risk of bias.


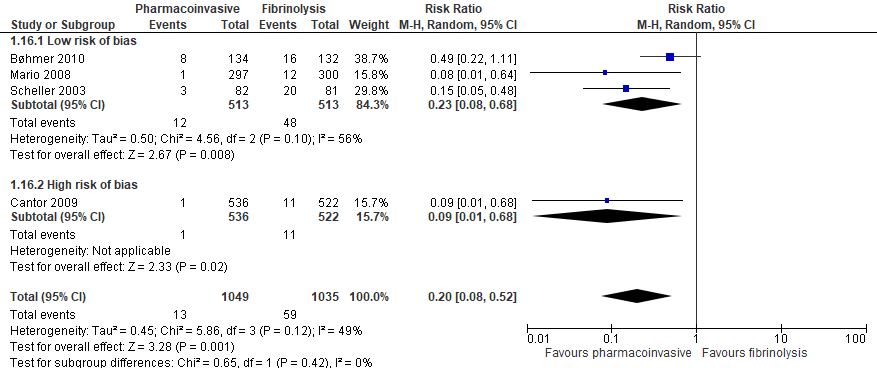


1. Recurrent ischemia (longest follow-up: 30 days to 12 months) by risk of bias.


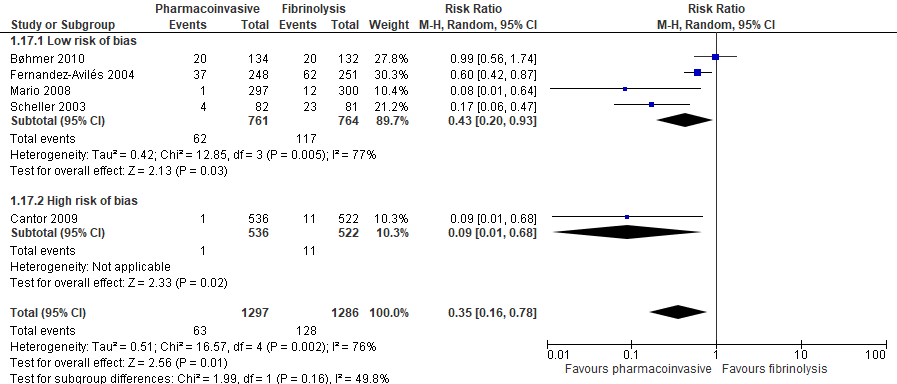

Supplement: S2 Fig — (DOCX) [file pone.0334309.s006.docx]
