# Supplementary material for: Pharmacoinvasive strategy versus fibrinolytic therapy alone in adults with ST-elevation myocardial infarction: A systematic review and meta-analysis
Source: PLoS One. 2025 Oct 9;20(10):e0334309. doi: 10.1371/journal.pone.0334309 (PMC12510495; doi:10.1371/journal.pone.0334309)

**Supplemental Figure 3. Sensitivity analysis excluding studies with high risk of bias.**

1. Mortality (follow-up: 30 days)


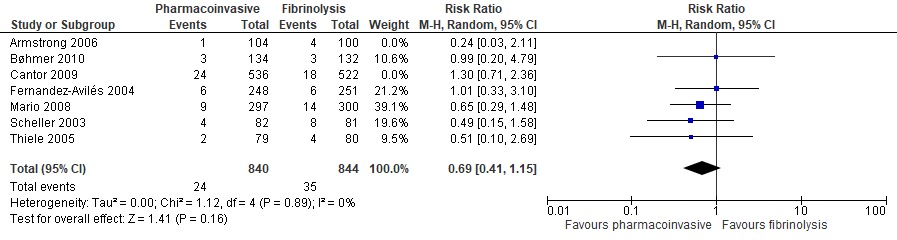


1. Mortality (longest follow-up: 30 days to 12 months)


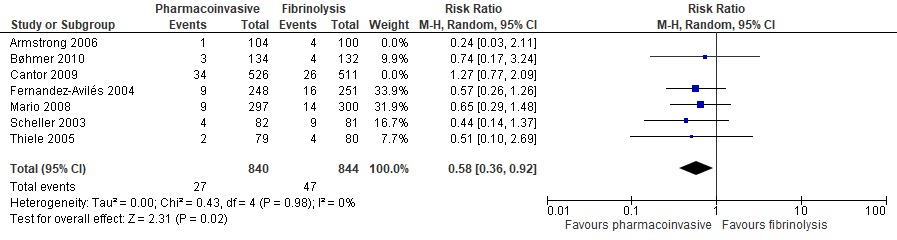


1. Reinfarction (follow-up: 30 days)


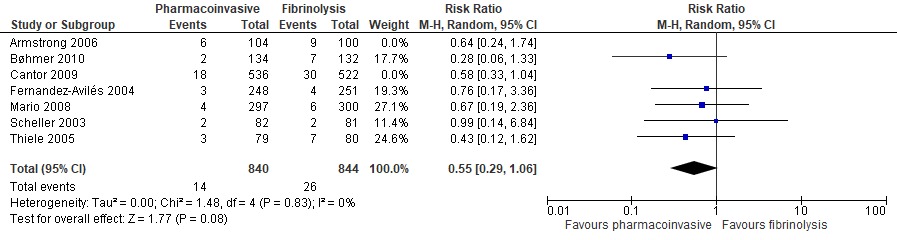


1. Reinfarction (longest follow-up: 30 days to 12 months)


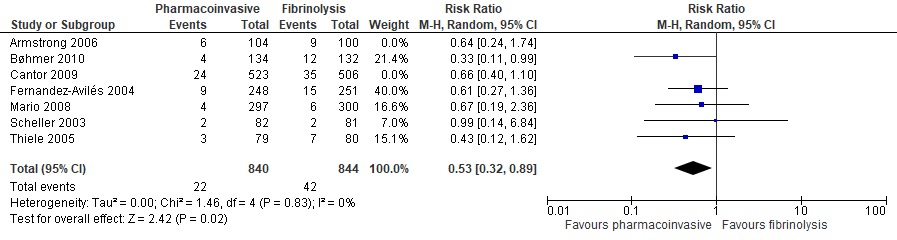


1. Major bleeding (follow-up: 30 days or during hospitalization)


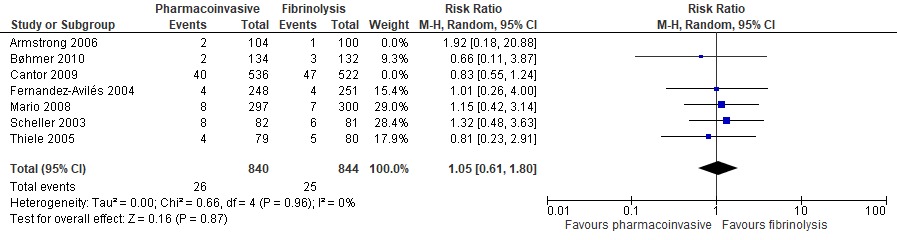


1. Stroke (follow-up: 30 days or during hospitalization)


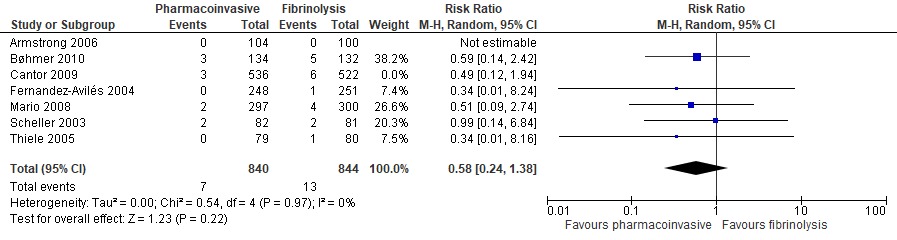


1. Recurrent ischemia (follow-up: 30 days)


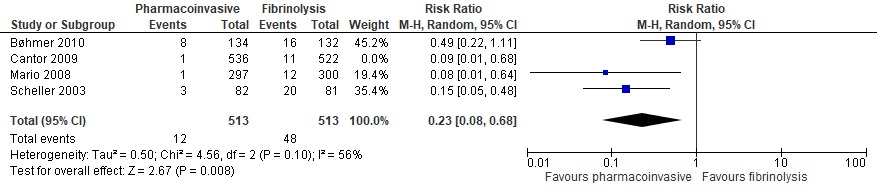


1. Recurrent ischemia (longest follow-up: 30 days to 12 months)


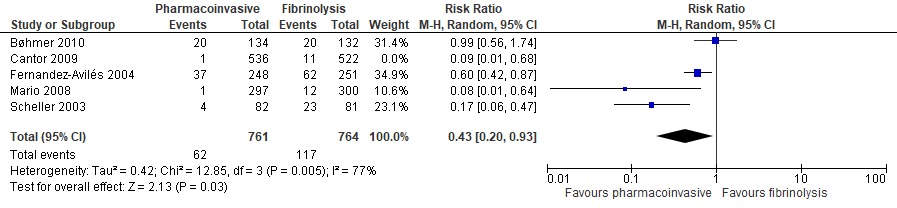


1. Cardiogenic shock


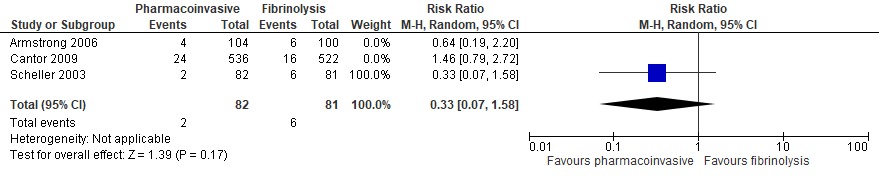

Supplement: S3 Fig — (DOCX) [file pone.0334309.s007.docx]
