# Supplementary material for: Pharmacoinvasive strategy versus fibrinolytic therapy alone in adults with ST-elevation myocardial infarction: A systematic review and meta-analysis
Source: PLoS One. 2025 Oct 9;20(10):e0334309. doi: 10.1371/journal.pone.0334309 (PMC12510495; doi:10.1371/journal.pone.0334309)
Supplement: Attachment — Submitted filename: Response to Reviewers.docx [file pone.0334309.s008.docx]

**Response to Reviewers**

**Title:** "Pharmacoinvasive Strategy versus Fibrinolytic Therapy in Adults with ST-Elevation Myocardial Infarction: A Systematic Review and Meta-Analysis"

| **Reviewer** | **No.** | **Comments** | **Localization** | **Response** |
| --- | --- | --- | --- | --- |
| **#1** | 1 | The prevalence of STEMI is stated to be "up to 40%" which may be misleading. Please clarify this statement. | Page 10, lines 52-54 | We appreciate the comment. Our intention was to emphasize the high burden of STEMI within acute coronary syndromes, so we have modified the text on lines 52 to 54 with the following: " STEMI is a public health problem in both industrialized and developing countries with an estimated global prevalence of over 3 million cases per year, making it the predominant acute manifestation of coronary heart disease”. |
| **#1** | 2 | The word "important" is repeatedly used for statistically significant differences. Please revise. | Multiple instances | We appreciate this observation. Following GRADE guidelines 26 and 32, the communication of results is based on absolute point effect and certainty of evidence. Because it is a minimally contextualized approach, the word important is used. <https://pubmed.ncbi.nlm.nih.gov/33857619/> <https://pubmed.ncbi.nlm.nih.gov/31711912/> |
| **#1** | 3 | Where no statistically significant difference is found, statements like "may have an important reduction" should be avoided. Consider discussing type II error instead. | Results and Discussion sections | Our phrasing follows GRADE 26 guidelines, which recommend basing conclusions on the absolute effect and certainty of the evidence rather than statistical significance. The interpretation of our results is aligned with these principles, and we are not considering type II error, as GRADE does not prioritize statistical significance in drawing conclusions. |
| **#1** | 4 | Some forest plots should be included in the main manuscript rather than the supplementary appendix. | Figures section | We agree and will include key forest plots for primary outcomes such as mortality and reinfarction in the main manuscript. |
| **#2** | 1 | Observational studies could provide insights into safety outcomes. Were they considered? | Methods section | Our focus was on RCTs due to their methodological robustness. However, we recognize that observational studies may offer valuable data on rare adverse events. To clarify this, we have added in lines 307- 309: “We excluded observational studies to focus on RCTs, which minimize bias and provide the most reliable evidence on efficacy and safety. However, we acknowledge that this may limit the detection of less frequent adverse events.” |
| **#2** | 2 | Justify the exclusion of databases such as Scopus, LILACS, and CINAHL. | Methods section | We appreciate this observation. Our database selection followed Cochrane's recommendations, which suggest prioritizing Medline (PubMed), Central and Embase, we also included ClinicalTrials.gov, and references from included studies. These databases provide comprehensive coverage of clinical trials. |
| **#2** | 3 | Even if statistical assessment of publication bias was not possible, was a qualitative evaluation performed? | Methods/Limitations | We acknowledge the importance of evaluating publication bias. A common indicator of this bias is the presence of small studies with positive results and funded by the industry. However, we did not find such characteristics among the included studies, suggesting that publication bias is unlikely to be a significant issue in our analysis. We added the following to lines 129–130: " The publication bias statistical assessment could not be performed due to the small number of studies per outcome, but there was no qualitative suspicion of publication bias." |
| **#2** | 4 | How were studies with high risk of bias handled? Were they excluded or included in sensitivity analyses? | Methods section | We conducted a sensitivity analysis excluding studies at high risk of bias. We added to the manuscript in lines 267–271: “In the sensitivity analysis excluding studies at high risk of bias, the results remained consistent with the primary analysis. The benefit of the intervention was more pronounced for outcomes such as mortality, myocardial infarction, and cardiogenic shock, though the overall conclusions were unchanged. For major bleeding, stroke, and angina, effect estimates were comparable to those observed in the main analysis.”  Corresponding sensitivity analysis forest plots have been included in the supplementary material. |
| **#2** | 5 | The threshold for "low risk of bias" (5/7 domains) is not standard. Justify its use. | Methods section | Thanks for the suggestion. We used the Cochrane Risk of Bias 1.0 tool, which lacks explicit guidance for general risk classification. This criterion was based on previous systematic reviews, such as the following:  <https://www.thelancet.com/journals/lancet/article/PIIS0140-6736(24)01357-6/fulltext#app-1> |
| **#2** | 6 | The definition of heterogeneity (I² <40% as "not important") differs from Cochrane guidelines. Please clarify. | Methods section | We revised our interpretation to align with Cochrane's classification, acknowledging that 0 - 40% represents “might not be important”.  <https://training.cochrane.org/handbook/current/chapter-10> |
| **#2** | 7 | How were MID thresholds determined? Were they based on literature, expert consensus, or empirical data? | Methods section | MID thresholds were based on literature and previous studies in cardiology. The article defining the MID thresholds is already cited in the review. |
| **#2** | 8 | The certainty of evidence for mortality was downgraded by two levels for imprecision. Justify why. | Table 2 & Discussion | The confidence interval includes both boundaries of the minimal important difference (MID), indicating high uncertainty regarding the true effect. This justifies downgrading the certainty of evidence by two levels.  GRADE 32: <https://www.jclinepi.com/article/S0895-4356(21)00108-6/fulltext> |
| **#2** | 9 | The time from fibrinolysis to PCI varied widely across studies. Was its impact assessed? | Discussion section | We acknowledge this concern. In lines 281 to 283 of the Discussion, we state that a meta-regression analysis could not be performed to assess the influence of treatment efficacy. |
| **#2** | 10 | The proportion of male participants varied significantly. Was gender-based response variability assessed? | Discussion section | A meta-regression analysis considering the percentage of male participants would have been ideal, but at least 10 studies are required for such an analysis, and our dataset did not meet this criterion. We did not conduct a subgroup analysis based on gender because all reported subgroup analyses from the included studies are presented in the "Subgroup Analysis" section of the results, no additional subgroups were available in the included studies. |
