# Supplementary material for: Pharmacoinvasive strategy versus fibrinolytic therapy alone in adults with ST-elevation myocardial infarction: A systematic review and meta-analysis
Source: PLoS One. 2025 Oct 9;20(10):e0334309. doi: 10.1371/journal.pone.0334309 (PMC12510495; doi:10.1371/journal.pone.0334309)
Supplement: Attachment — Submitted filename: Response_to_Reviewers_auresp_2.docx [file pone.0334309.s009.docx]

**Response to Reviewers**

**Title:** Pharmacoinvasive strategy versus fibrinolytic therapy alone in adults with ST-elevation myocardial infarction: a systematic review and meta-analysis

We thank the editors and reviewers for their thorough and constructive feedback. Below we provide a point-by-point response to each comment, along with a summary of the corresponding revisions made in the manuscript.

| **Reviewer** | **No.** | **Comments** | **Localization** | **Response** |
| --- | --- | --- | --- | --- |
| **#2** | 1 | The authors acknowledged the impossibility of performing a statistical assessment of publication bias due to the limited number of studies, and added in lines 129-130 that no qualitative suspicion of bias was observed. This revision is constructive; however, the qualitative assessment remains limited, as it was based solely on the absence of small industry-funded studies. | Methods | We appreciate the observation. According to GRADE Guidelines 5, “it is very difficult to be confident that publication bias is absent, and almost equally difficult to know where to place the threshold and rate down for its likely presence. Recognizing these challenges, the term GRADE suggests using in evidence profiles for publication bias is ‘undetected.’” Following this recommendation, we have modified the corresponding paragraph in the manuscript to: “*The publication bias statistical assessment could not be performed due to the small number of studies per outcome, but publication bias was undetected based on a qualitative evaluation of small trials with positive results and industry-funded studies*.” |
| **#2** | 2 | The criterion of considering “low risk of bias” as meeting 5 out of 7 domains was justified by referencing previous systematic reviews, with an appropriate citation (p. 15, lines 116-122), thus demonstrating alignment with established methodological precedents. Nonetheless, the authors’ response and the manuscript fail to discuss the relevance of critical domains such as random sequence generation or allocation concealment, whose qualitative weighting could influence the overall risk classification. | Methods | We agree with the suggestion and additionally considered the qualitative implications of the risk of bias according to the importance of its items.  We clarified this in the Methods: “*We considered a study to be at low risk of bias if it had at least 5 low risk items, following criteria used in previous systematic reviews [14]. However, we also considered the qualitative importance of individual domains. Deficiencies in these domains were considered in the interpretation of results, regardless of the overall score*.”  and in the Discussion: “*Although most domains of the RoB 1.0 tool were judged as low risk in the included studies, several critical aspects should be considered when interpreting our findings. The most frequent limitation was unclear allocation concealment in 5 of the 7 studies, which could lead to selective enrollment of participants, baseline imbalances, and overestimation of the observed treatment effects. In addition, lack of blinding of participants and personnel was observed in 4 of the 7 studies. For subjective outcomes (e.g., angina), this lack of blinding could result in differences in expectations and decision-making by both participants and physicians. However, the critical outcomes in the present study are less prone to subjective influence*.” |
| **#2** | 3 | The correction to the definition of heterogeneity to align with Cochrane standards (I² 0–40% as “might not be important”) demonstrates responsiveness and adherence to current guidelines. However, the manuscript does not explain why outcomes with substantial heterogeneity – such as long-term angina (I² = 76%, p. 58, lines 241–247) – did not result in a downgrade of the certainty of evidence within the GRADE assessment (Table 2, p. 56). | Methods and results | We appreciate the observation. In the case of long-term angina (I² = 76%), although the heterogeneity was statistically substantial, the direction of effect was consistent across studies, with all point estimates favoring the pharmacoinvasive strategy. According to GRADE guidance, inconsistency warrants downgrading when variability in results is both substantial and unexplained in a way that could alter the interpretation of the effect. We clarify this aspect in the certainty of the evidence section: “*Regarding heterogeneity, we rated down one level if I2 was between 40-80% with inconsistency of the direction of the association and rated down two levels if I2 was >80% with inconsistency of the direction of the association*.” |
| **#2** | 4 | The justification of MIDs was based on the literature, with citation of reference [19] (Armstrong et al., 2013; p. 65, line 137), a clinical trial comparing fibrinolysis and primary PCI, in an effort to align with GRADE guidance. However, reference [19] does not explicitly define MIDs or describe the process for determining them, and neither the response to the reviewer nor the manuscript (p. 16, lines 142–144) provides sufficient detail on how MID values were selected or derived. While data from [19] (e.g., a 1.9% difference in the composite outcome) may have been used to infer MID thresholds, the lack of transparency regarding this derivation process compromises methodological clarity. | Methods | We appreciate the observation, we have detailed the process we followed to establish MIDs in Methods: “*To assess imprecision, we followed the GRADE guidelines 34, which recommend establishing minimally important differences (MID) for each outcome.[19] To define the MIDs, we first searched PubMed for studies that specified these values for the target population; however, no such studies were identified. The search strategy is detailed in Supplementary Table 3. Consequently, we determined the MIDs for each outcome based on the results of a trial that compared the most effective intervention for STEMI patients (primary PCI) with fibrinolysis alone (the comparator group in the present study). [20] For outcomes not specified in that trial, the MIDs were determined by consensus among the authors of the present study*.” |
| **#3** | 1 | The title implies a direct comparison between PS and fibrinolysis, but the PS intervention includes fibrinolysis followed by PCI. This could mislead readers into thinking PS excludes fibrinolysis (Page 1, Title). | Title & Abstract | To avoid ambiguity, we have revised the title to clarify that the comparator is fibrinolysis alone. The title now reads: “*Pharmacoinvasive strategy versus fibrinolytic therapy alone in adults with ST-elevation myocardial infarction: a systematic review and meta-analysis*.” |
| **#3** | 2 | No mention of registration (PROSPERO) or risk of bias assessment tool (Page 1, Abstract). | Abstract | We agree with this observation, and we modified the Methods section of the abstract to read as follows:  “*The review protocol was registered in PROSPERO (CRD42022309130). We included randomized controlled trials (RCTs), assessed risk of bias with the Cochrane Risk of Bias 1.0 tool, and calculated pooled relative risks and mean differences*.” |
| **#3** | 3 | Absolute effects lack context without baseline risks (Page 1, Lines 35–36). | Abstract | We have added in abstract the absolute effects together with baseline risks for context. |
| **#3** | 4 | The statement "PS has trivial or no effect on major bleeding" (Line 41) is overstated; the CI (-17 to +13) includes both trivial harm and benefit, making "no effect" speculative. | Abstract | According to GRADE, conclusions should not rely solely on statistical significance, as this reflects only one aspect of the certainty of evidence: imprecision. The minimally contextualized GRADE approach focuses on whether the estimated effect is minimally important, based on the position of the point estimate in relation to the MID thresholds. The final phrasing of results depends on both the location of the point estimate and the certainty of evidence (high, moderate, low, or very low). This approach is described in GRADE guideline 26 and, more specifically for systematic reviews with minimally contextualized approach, in guideline 34. For the outcome of revascularization, considering a MID of ±17, the 95% confidence interval (-17 to +13) marginally overlaps the lower threshold. Therefore, we decided not to downgrade the certainty for imprecision. |
| **#3** | 5 | The abstract claims "high certainty" for reinfarction but omits downgrading reasons for other outcomes (Page 1, Lines 35–46). | Abstract | We appreciate this observation. Due to the journal’s word limit for the abstract (300 words), it was not possible to include all downgrading reasons for each outcome. The full GRADE assessment, including downgrading reasons for all other outcomes is detailed in the results section. |
| **#3** | 6 | Conclusion: States PS "may have an important reduction of mortality and stroke" but does not clarify that CIs include null effects (Mortality 95% CI: -16 to +10; Stroke: -10 to +2) (Page 1, Lines 44–46). | Abstract | According to GRADE, conclusions should not rely solely on statistical significance, as this reflects only one aspect of the certainty of evidence: imprecision. The minimally contextualized GRADE approach focuses on whether the estimated effect is minimally important, based on the position of the point estimate in relation to the MID thresholds. The final phrasing of results depends on both the location of the point estimate and the certainty of evidence (high, moderate, low, or very low). This approach is described in GRADE guideline 26 and, more specifically for systematic reviews with minimally contextualized approach, in guideline 34. |
| **#3** | 7 | Fails to justify why PS (fibrinolysis plus PCI) is compared to fibrinolysis alone instead of primary PCI, the gold standard (Page 13, Lines 66–72). | Introduction | We agree with your observation, we have modified the introduction: “*As a result, fibrinolysis alone remains the most commonly available and feasible reperfusion strategy in these settings. In this context, the pharmacoinvasive strategy combines early fibrinolysis followed by routine coronary angiography and potential PCI within 2 to 24 hours and emerges as a practical and guideline-endorsed alternative when timely PCI cannot be performed [5,8,9]. Importantly, while primary PCI is the gold standard of treatment, it is important to compare the pharmacoinvasive strategy with fibrinolysis alone because this reflects a more realistic and context-appropriate clinical decision pathway in resource-limited environments, where the majority of patients do not have access to timely primary PCI [10,11]*” |
| **#3** | 8 | The response to Reviewer #1 (Page 2, Lines 1–3) revised STEMI prevalence to "over 3 million cases per year," yet the manuscript retains "prevalence that can reach up to 40%" (Page 46, Line 54). | Introduction | The phrase “*prevalence that can reach up to 40%*” has been removed to avoid confusion. The manuscript now states: “*STEMI is a public health problem… with an estimated global prevalence of over 3 million cases per year*.” |
| **#3** | 9 | The objective ("to compare PS versus fibrinolysis") is vague; it should specify efficacy/safety outcomes (Page 14, Line 77). | Introduction | We agree. The objective has been revised to specify the efficacy and safety outcomes assessed: " *the objective of this systematic review (SR) is to assess the efficacy and safety of pharmacoinvasive strategy compared with fibrinolysis in adults with ST-segment elevation myocardial infarction. The outcomes assessed included mortality, reinfarction, recurrent ischemia, need for revascularization, length of hospital stay, stroke, major bleeding, heart failure, and cardiogenic shock*." |
| **#3** | 10 | "Primary PCI performed 2–24 hours after fibrinolysis" (Page 14, Line 85) is ambiguous. Does "primary PCI" imply immediate PCI, conflicting with the 2–24h window? | Methods | The term “primary” has been removed. The sentence now reads: “*PCI performed over a period of time of 2 to 24 hours from the end of fibrinolysis administration*”. |
| **#3** | 11 | "Cardiac failure," "angina," and "stroke" lack standardized diagnostic criteria (Page 14, Lines 88–92). | Methods | We have revised and clarified the definitions of the specified outcomes to ensure consistency with the terminology and criteria used in the included studies. Specifically, we changed the term "angina" to "recurrent ischemia", which better reflects the definitions applied across trials. The definitions of heart failure and stroke remained consistent and required no modification. Below, we provide a summary of how these outcomes were defined in the included studies:  Heart failure  Armstrong 2006: (3) Congestive heart failure: (i) Physician’s decision to treat CHF with a diuretic, intrave nous inotropic agent or intravenous vasodilator and either (a) the presence of pulmonary edema or pulmonary vascu lar congestion on chest X-ray believed to be of cardiac cause or (b) at least two of the following: (1) rales greater than one-third up the lung fields believed to be due to CHF. (2) PCWP .18mmHg (3) Dyspnoea, with documented pO2 less than 80 mmHg on room air or O2 saturation ,90% on room air, without significant lung disease.  Cantor 2009: heart failure that required treatment 6 hours or more after enrollment and either pulmonary ede ma on a chest radiograph, rales, or a pulmonary capillary wedge pressure greater than 18 mm Hg.  Angina  Bohmer 2010: unstable angina (chest pain at rest suspicious for coronary disease with or without ECG changes), recurrent angina grade II to IV (Canadian Cardiovascular Society classification)  Cantor 2009: Recurrent ischemia was defined as chest pain lasting 5 minutes or longer associated with ST-segment or T-wave changes  Mario 2008: recurrent chest pain with ST-segment deviation or defi nite T-wave inversion occurring more than 12 h after randomisation persisting for at least 10 min despite administration of nitrates, β blockers, or calcium channel blockers and not fulfilling the diagnosis of myocardial reinfarction.  Scheller 2003: recurrent angina pectoris lasting for more than 15 min despite the administration of nitrates or being accompanied by electrocardiographic changes, pulmonary edema, or hypotension  Fernandez-Aviles 2004: spontaneous (at rest) or stress-induced recurrence of typical angina pectoris (or anginal equivalent) that had to coincide with new ECG abnormalities, or abnormal stress test  Stroke:  Armstrong 2006: Stroke after thrombolysis  Bohmer 2010: new focal, neurological deficit of vascular origin lasting more than 24 h.  Cantor 2009: intracranial hemorrhage  Fernandez-Aviles 2004: intracranial hemorrhage  Mario 2008: intracranial hemorrhage  Scheller 2003: Stroke after thrombolysis  Thiele 2005: fatal stroke or stroke causing significant mental or physical handicap |
| **#3** | 12 | Database Justification: No rationale for excluding Scopus/LILACS/CINAHL despite Reviewer #2's query (Page 15, Lines 96–99; Page 2, Response #2). | Methods | According to the Cochrane Handbook for Systematic Reviews of Interventions, it is recommended that “CENTRAL and MEDLINE, together with Embase (if access to Embase is available to the review team), should be searched for all Cochrane Reviews.” Furthermore, the Handbook also highlights that “searches for studies should be as extensive as possible to reduce the risk of publication bias and to identify as much relevant evidence as possible,” and encourages the inclusion of topic-specific and regional databases, such as CINAHL for nursing-related topics or APA PsycInfo for psychological interventions, as well as regional resources like LILACS.  In line with these recommendations, we conducted a comprehensive search in CENTRAL, MEDLINE, and Embase. Additionally, we included Web of Science, which indexes a broad range of journals, including those from regional databases such as SciELO. |
| **#3** | 13 | The "diriment author" (Page 15, Line 105) role is not defined. | Methods | We appreciate the reviewer’s observation. We clarified this point in the manuscript by specifying that the “diriment author” acted as a third independent reviewer responsible for adjudicating disagreements. |
| **#3** | 14 | Using a fixed threshold (5/7 low-risk domains = "low RoB") contradicts Cochrane guidelines, which discourage numerical thresholds (Page 15, Lines 121–122; Page 3, Response #5). | Methods | We appreciate this observation. Cochrane and GRADE do not provide an explicit numerical threshold to classify overall risk of bias. To operationalize our assessment, we adopted the 5/7 domain criterion as applied in previous systematic reviews. Nonetheless, our judgments also considered the qualitative implications of the identified biases, ensuring consistency with GRADE guidance.  <https://www.cochrane.org/authors/handbooks-and-manuals/handbook/current/chapter-08#section-8-2-4> |
| **#3** | 15 | Figure 2 (Page 35) uses "+" and "○" without a legend. | Methods | We added a legend in Table 2:  ⨁⨁⨁⨁ High certainty: We are very confident that the true effect lies close to that of the estimate of the effect.  ⨁⨁⨁◯ Moderate certainty: We are moderately confident in the effect estimate: The true effect is likely to be close to the estimate of the effect, but there is a possibility that it is substantially different  ⨁⨁◯◯ Low certainty: Our confidence in the effect estimate is limited: The true effect may be substantially different from the estimate of the effect.  ⨁◯◯◯ Very Low certainty: We have very little confidence in the effect estimate: The true effect is likely to be substantially different from the estimate of effect |
| **#3** | 16 | Defining I² <40% as "not important" conflicts with Cochrane (30–60% = moderate heterogeneity) (Page 49, Line 127; Page 3, Response #6). | Methods | We appreciate this observation. We have revised our interpretation to ensure alignment with the Cochrane Handbook for Systematic Reviews of Interventions, which provides the following guidance for interpreting heterogeneity based on the I² statistic:  0% to 40%: might not be important;  30% to 60%: may represent moderate heterogeneity*;  50% to 90%: may represent substantial heterogeneity*;  75% to 100%: considerable heterogeneity*.  *Interpretation depends on context and consistency across studies  (Source: https://training.cochrane.org/handbook/current/chapter-10)  In our review, we considered heterogeneity below 40% as not important, in accordance with this guidance. It is important to note that the only outcome for which the certainty of the evidence was downgraded due to heterogeneity was cardiogenic shock, where the I² was 47% and the direction of effect estimates varied across studies. Thus, whether a threshold of 30% or 40% is used would not have changed the overall interpretation of the results. |
| **#3** | 17 | Minimal important differences (MIDs) for outcomes (mortality: ±2/1000) lack justification. No citation or method for deriving MIDs is provided (Page 22, Lines 192–194; Page 3, Response #7). | Methods | We appreciate the observation, we have detailed the process we followed to establish MIDs in Methods: “*To assess imprecision, we followed the GRADE guidelines 34, which recommend establishing minimally important differences (MID) for each outcome.[19] To define the MIDs, we first searched PubMed for studies that specified these values for the target population; however, no such studies were identified. The search strategy is detailed in Supplementary Table 3. Consequently, we determined the MIDs for each outcome based on the results of a trial that compared the most effective intervention for STEMI patients (primary PCI) with fibrinolysis alone (the comparator group in the present study). [20] For outcomes not specified in that trial, the MIDs were determined by consensus among the authors of the present study*.” |
| **#3** | 18 | Qualitative assessment ("no suspicion of bias") is inadequate without funnel plots or registry searches (Page 49, Lines 128–129). | Methods | We appreciate the observation. According to GRADE Guidelines 5, “it is very difficult to be confident that publication bias is absent, and almost equally difficult to know where to place the threshold and rate down for its likely presence. Recognizing these challenges, the term GRADE suggests using in evidence profiles for publication bias is ‘undetected.’” Following this recommendation, we have modified the corresponding paragraph in the manuscript to: “*The publication bias statistical assessment could not be performed due to the small number of studies per outcome, but publication bias was undetected based on a qualitative evaluation of small trials with positive results and industry-funded studies*.” |
| **#3** | 19 | Pre-specified subgroups ("bled at time of fibrinolysis") are not supported by included studies, yet this is not acknowledged (Page 50, Lines 134–135). | Methods | We have clarified in the manuscript that the included studies did not report results stratified by these subgroups, which precluded performing the analyses as originally planned in the protocol. “*We pre-specified subgroup analysis according to risk of bias. We could not perform pre-specified subgroup analysis according to stent types, age, comorbidities, bled at the time of fibrinolysis, success of the fibrinolytic therapy alone, and history of myocardial infarction episode because the included studies did not evaluate or report outcomes stratified by these subgroups*.” |
| **#3** | 20 | Table 1 Errors:  "Time from fibrinolysis to PCI" for GRACIA-1 is listed as "16.7 hours (5.6)" without units (Page 20). | Results | The error has been corrected, and the time from fibrinolysis to PCI in GRACIA-1 is now presented in minutes with appropriate units. |
| **#3** | 21 | Table 1 Errors:  Rescue PCI rates in the fibrinolysis group (14–34.9%) are reported but not contextualized (Page 17, Line 180). | Results | We agree with this comment, we added the following to the discussion section: “*Another important finding is that, among patients who received fibrinolysis alone, the need for rescue PCI ranged from 14.0% to 34.9% across studies. These findings highlight that a considerable proportion of patients may not achieve adequate reperfusion and ultimately require urgent invasive intervention, underscoring the potential benefits of a pharmacoinvasive strategy*.” |
| **#3** | 22 | 4/7 studies had high RoB in ≥1 domain, but sensitivity analysis (Page 26, Lines 257–261) was limited to supplemental material, not main text. | Results | We acknowledge the reviewer’s comment, but we think that including all sensitivity analysis figures in the main text would have resulted in excessive length; therefore, they were placed in the supplemental material (Supplemental Figure S7). However, the main findings of the sensitivity analyses are explicitly summarized in the Results section, Sensitivity analysis, ensuring that the key information is available in the main manuscript. |
| **#3** | 23 | Revascularization outcomes (30-day/longest) are labeled "high certainty" despite being based on only 2–3 studies (Page 22, Table 2). | Results | The assessment of the certainty of the evidence is based on criteria such as risk of bias, heterogeneity, publication bias, indirectness, and imprecision. Following the minimally contextualized approach outlined in GRADE Guidelines 34, the evaluation of imprecision considers the relationship between the 95% confidence interval (CI) and the minimally important differences (<https://doi.org/10.1016/j.jclinepi.2022.07.014>) . In this case, the 95% CI for the outcome of revascularization does not cross the predefined MID threshold (±30 events), neither at 30 days nor at the longest follow-up. Therefore, the certainty of the evidence should not be downgraded due to imprecision. |
| **#3** | 24 | Angina at longest follow-up had I²=76% but was not downgraded for inconsistency (Page 25, Line 251). | Results | Although I² was 76%, all point estimates favored the pharmacoinvasive strategy indicating consistency in the direction of the effect. Therefore, despite statistical heterogeneity, we did not downgrade the certainty due to inconsistency, following GRADE criteria. |
| **#3** | 25 | Mortality is downgraded twice for imprecision (RR 0.87, 95% CI: 0.59–1.27), but the Discussion claims PS "may have an important reduction" (Page 23, Table 2; Page 27, Line 271). | Results | As noted in a previous comment, the final phrasing of results depends on both the location of the point estimate relative to the minimally important difference and the certainty of the evidence (high, moderate, low, or very low). This approach is outlined in GRADE Guideline 26 and, more specifically for systematic reviews using the minimally contextualized approach, in GRADE Guideline 34.  In this case, since the certainty of the evidence is low, we use the term “may,” and because the point estimate lies beyond the MID threshold, we consider the effect to represent an important reduction. |
| **#3** | 26 | Highlights PS benefits but downplays null findings (major bleeding: RR 0.91, 95% CI: 0.66–1.26) (Page 27, Lines 276–278). | Discussion | As detailed in a previous comment, according to GRADE, conclusions should not rely solely on statistical significance, as this reflects only one aspect of the certainty of evidence: imprecision. The minimally contextualized GRADE approach focuses on whether the estimated effect is minimally important, based on the position of the point estimate in relation to the MID thresholds. The final phrasing of results depends on both the location of the point estimate and the certainty of evidence (high, moderate, low, or very low). |
| **#3** | 27 | Studies were from high-income countries (Canada, Europe), but implications are extended to LMICs without caveats (Page 28, Line 285). | Discussion | We agree with this comment we added the following to the discussion: “*Unfortunately, low- and middle-income countries account for approximately 80% of the global population, and access to the treatment of choice (primary PCI) is often limited [5]. This limitation is especially pronounced in rural and underserved areas, where specialized centers are scarce and timely transportation is frequently not feasible. Moreover, findings from studies conducted in high-income countries may not be directly applicable to low-middle income countries due to substantial differences in healthcare infrastructure, availability of trained personnel, emergency response systems, comorbidity burden, and sociodemographic characteristics. These differences can influence both the effectiveness and safety of therapeutic strategies such as the pharmaco-invasive approach*.” |
| **#3** | 28 | Limitations  Time-to-PCI Variability: The impact of widely varying fibrinolysis-to-PCI times (135 min–16.7 hours) on outcomes is not discussed (Page 28, Lines 293–295). | Discussion | We agree with this comment, we added the following to the discussion: “*The time between fibrinolysis and PCI ranged from 135 minutes (2.25 hours) to 1002 minutes (16.7 hours). However, due to the limited number of studies available, we were unable to perform a meta-regression analysis to determine whether time had any influence on treatment efficacy. Nonetheless, evidence from observational studies suggests that performing PCI as early as possible within the pharmacoinvasive strategy is associated with greater clinical benefit [34*].” |
| **#3** | 29 | Despite known gender disparities in STEMI, subgroup analysis was not performed due to "insufficient studies," but TRANSFER-AMI reported gender-specific data (Page 26, Lines 263–268; Page 4, Response #10). | Discussion | Thanks for the observation, we detailed this aspect in the results section: “*Bhan et al. in the TRANSFER-AMI trial compared the effect of the pharmacoinvasive strategy in men and women, but found no difference between the two groups for mortality, reinfarction, recurrent ischemia, heart failure, cardiogenic shock, transient ischemic attack/stroke and bleeding at 30 days.[31*]” |
| **#3** | 30 | Claims PS reduces mortality and stroke despite low certainty and CIs including null effects (Page 30, Lines 348–351). | Conclusion | As detailed in a previous comment, according to GRADE, conclusions should not rely solely on statistical significance, as this reflects only one aspect of the certainty of evidence: imprecision. The minimally contextualized GRADE approach focuses on whether the estimated effect is minimally important, based on the position of the point estimate in relation to the MID thresholds. The final phrasing of results depends on both the location of the point estimate and the certainty of evidence (high, moderate, low, or very low). |
| **#3** | 31 | Does not address uncertain evidence for cardiac failure/cardiogenic shock (very low certainty). | Conclusion | We added the results of heart failure and cardiogenic shock to the conclusion: “*In* *this systematic review, we included 7 RCTs involving a total of 3053 patients. Compared to fibrinolysis, the pharmacoinvasive strategy has an important reduction on reinfarction, revascularization, and angina; probably has an important reduction on mean hospital stay length; and may have an important reduction of mortality and stroke. Also, PS may reduce cardiac failure and cardiogenic shock, but the evidence is very uncertain. In addition, the risk of major bleeding did not increase*.” |
